# Supplementary material for: Inhibitory Mechanism of Quercimeritrin as a Novel α-Glucosidase Selective Inhibitor
Source: Foods. 2023 Sep 13;12(18):3415. doi: 10.3390/foods12183415 (PMC10528180; doi:10.3390/foods12183415)
Supplement: Supplementary file 1 [file foods-12-03415-s001.zip › foods-2578693-supplementary.pdf]

Appendix Supplementary data

Table S1

α-Glucosidase and α-amylase inhibitory efficacy.

compound	α-glucosidase inhibition IC50±SEM (μM)	α-amylase inhibition IC50±SEM (μM)
Avicularin	>500	>500
Apigetrin	187.22±6.93	508.57±86.99
Catechin gallate	7.53±0.55	148.61±33.57
Epicatechin gallate	>250	>250
Hyperoside	>500	>500
Isovitexin	>500	>500
Oroxin A	>500	>250
Quercituron	>500	>500
Quercitrin	>500	>500
Quercimeritrin	79.88±7.89	>250
Quercetagitrin	>250	>125
Trifolin	>250	>500
Acarbose	584.56±25.16	22.07±3.64

Note: IC50 values (mean ± standard error of the mean); Acarbose: Standard inhibitor for glucosidase and amylase.

Table S2

*V*max and *K*m values of Quercimeritrin inhibiting α-amylase and α-glucosidase.

[I] /μM	α-glucosidase				α-amylase			
	Vmax	R	Km	R	Vmax	R	Km	R
500	0.1761	0.0100	1.9577	0.2826	0.0508	0.0007	3.7194	0.1491
250	0.1890	0.0078	1.5085	0.1825	0.0503	0.0013	2.8889	0.1394
125	0.1916	0.0081	1.1322	0.0998	0.0526	0.0014	1.7999	0.0502
62.5	0.2000	0.0179	0.8040	0.0981	0.0522	0.0018	1.3355	0.0401
31.3	0.2028	0.0150	0.5761	0.0386	0.0532	0.0020	0.9820	0.0216
15.6	0.2151	0.0378	0.5011	0.0636	0.0521	0.0038	0.7646	0.0272
0	0.2188	0.0294	0.3589	0.0228	0.0506	0.0025	0.6294	0.0164

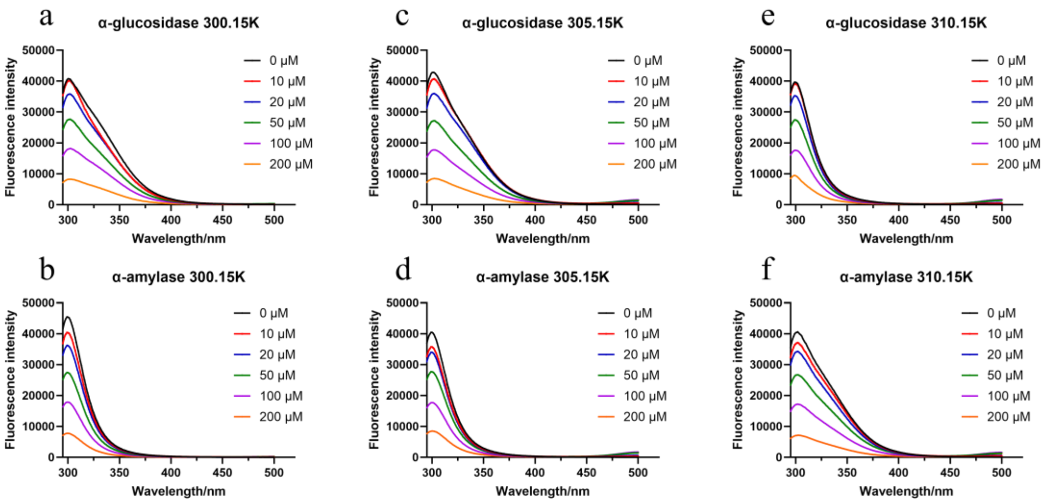

Figure S1. (a) and (b) Fluorescence spectra of α-glucosidase and α-amylase in the presence of Quercimeritrin at various concentrations (300.15 K); (c) and (d) Fluorescence spectra of α-glucosidase and α-amylase in the presence of Quercimeritrin at various concentrations (305.15 K); (e) and (f) Fluorescence spectra of α-glucosidase and α-amylase in the presence of Quercimeritrin at various concentrations (310.15 K).

Table S3

Quenching constants (*K*sv), binding constants (*K*a), and thermodynamic parameters of Quercimeritrin with α-glucosidase and α-amylase interaction at different temperatures.

System	T (K)	Ksv (10^5 /M)	R	Kq $(M·S)^{-1}$	n	Ka (10^5 /M)	R
	300.15	0.17 ±0.03	0.99	$1.7×10^{12}$	1.35	8.71 ±1.23	0.99
α-glucosidase	305.15	0.19 ±0.02	0.99	$1.9×10^{12}$	1.20	1.04 ±0.24	0.99
	310.15	0.21 ±0.04	0.99	$2.1×10^{12}$	1.09	0.41 ±0.06	0.99
	300.15	0.13 ±0.03	0.98	$1.3×10^{12}$	1.20	0.66 ±0.02	0.99
α-amylase	305.15	0.15 ±0.02	0.99	$1.5×10^{12}$	1.10	0.35 ±0.01	0.99
	310.15	0.18 ±0.04	0.99	$1.8×10^{12}$	1.04	0.26 ±0.01	0.99

Table S4

The thermodynamic parameters ΔH, ΔS, and ΔG of Quercimeritrin with α-glucosidase and α-amylase interaction at different temperatures.

System	T (K)	ΔH (kJ/mol)	ΔG (KJ/mol)	ΔS (J/mol/K)
	300.15	-237 ±23.42	-34.1 ±9.28	
α-glucosidase	305.15		-29.3 ±9.37	-677.34 ±87.38
	310.15		-27.4 ±7.84	
	300.15		-0.28 ±0.04	
α-amylase	305.15	-14.10 ±3.89	-0.27 ±0.02	-153.31 ±34.89
	310.15		-0.26 ±0.06	

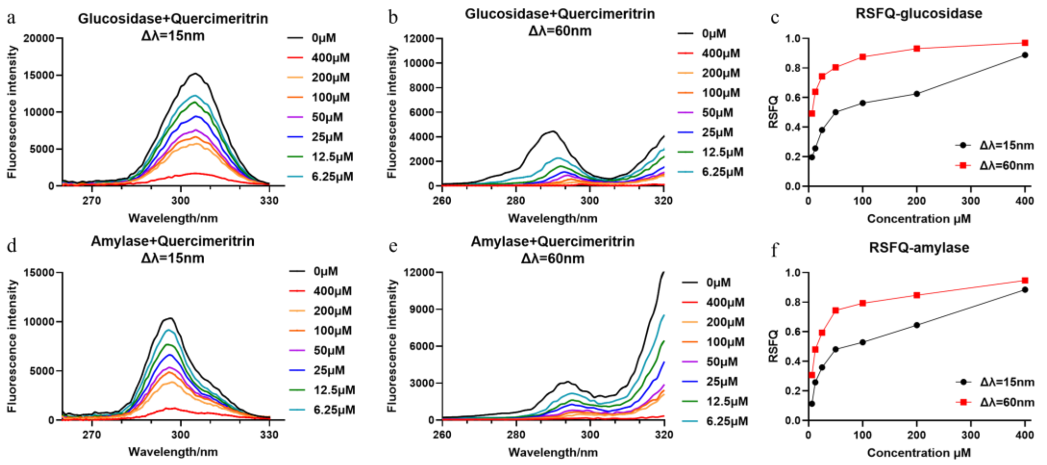

Figure S2. Synchronous fluorescence spectra of α-glucosidase and α-amylase with Quercimeritrin at Δλ = 15 nm and Δλ = 60 nm (a), (b), (d), (e), the corresponding plot of the ratios of synchronous fluorescence quenching (RSFQ) (c), (f).

Table S5

Molecular docking scores of inhibitor and acarbose.

Ligand with enzyme	Grid Score(kcal/mol)	Grid_vdw(kcal/mol)	Grid_es(kcal/mol)
Quercimeritrin with α-glucosidases	-83.167130	-53.928558	-29.238567
Quercimeritrin with α-amylase	-50.228935	-39.985130	-10.243806
Acarbose with α-glucosidases	-63.452332	-46.350677	-17.101656
Acarbose with α-amylase	-95.144455	-70.393715	-24.750744

Note: Grid_es denotes the grid score for electrostatic force, whereas Grid_vdw denotes the grid score for van der Waals force. Grid_vdw and Grid_es were added to determine the values of grid scores.

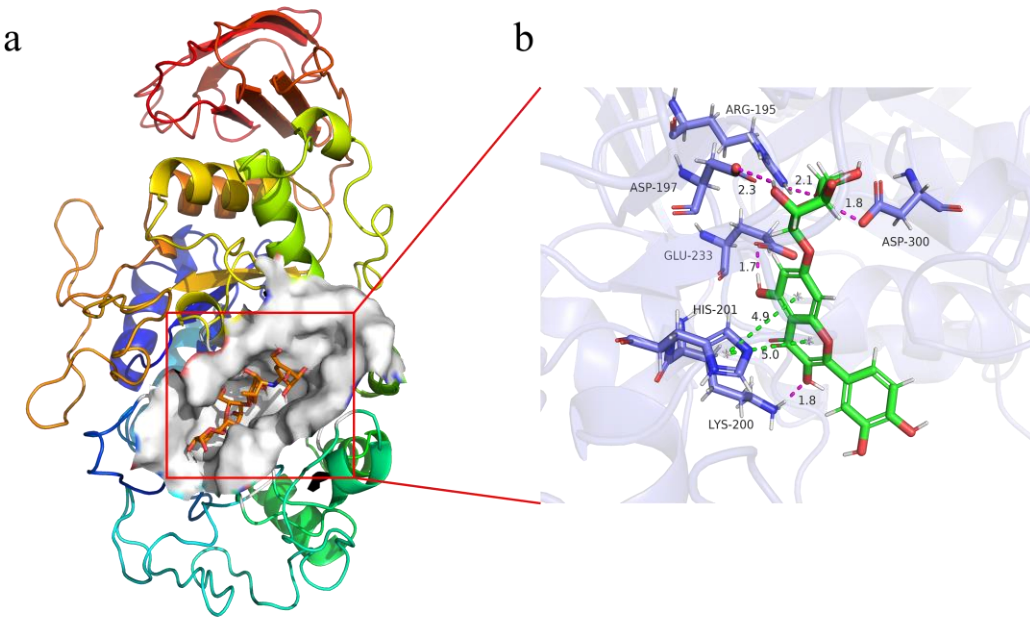

Figure S3. The way the compound binds to glucosidase. (a) An overall diagram of the hydrophobic pocket and active site interaction of the compound with α-glucosidase, (b) three-dimensional binding of the compound with α-glucosidase. Labeled key residues, hydrogen bonds formed between ligands and key residues (purple), and π–π interactions (green) are shown as dotted or solid lines.

Table S6

Virtual screening result.

PubChem ID	Ligand scores -2QMJ	Ligand scores -1CXW
74819354	-10.6	-10.9
107905 (Epicatechin gallate)	-8.6	-7.5
193124	-8.4	-8.4
11972399	-8.4	-9
14135335	-8.4	-8.8
199472	-8.3	-7.6
6419835 (Catechin gallate)	-8.3	-7.4
21580051	-8.3	-8
21626477	-8.3	-8.3
155289773	-8.3	-8.3
64982	-8.3	-8.9
5281675	-8.2	-7.1
5320861	-8.2	-8
14655552	-8.2	-8.5
91827018	-8.2	-8.5
185766	-8.1	-8.8
441699	-8.1	-7.5
5320313 (Oroxin A)	-8.1	-8.8
5748205	-8.1	-8.5

15540754	-8.1	-8.6
21629877	-8.1	-8
101686456	-8.1	-9
5274585 (Quercituron)	-8	-7.1
5282160 (Quercimeritrin)	-8	-6.8
5319484	-8	-8.7
11641481	-8	-8.2
443650	-7.9	-7.1
5280441	-7.9	-7.2
5280704 (Apigetrin)	-7.9	-8.6
5281757	-7.9	-8.5
5280459 (Quercitrin)	-7.8	-7
5317471	-7.8	-7.8
5320623	-7.8	-8.7
5316673	-7.7	-6.2
5321577	-7.7	-6.4
5490064 (Avicularin)	-7.7	-7.9
11016019	-7.7	-8.7
74079809	-7.7	-7.8
5281643 (Hyperoside)	-7.6	-6.6
5282149 (Trifolin)	-7.6	-6.7
5320826 (Quercetagitrin)	-7.6	-8
6455477	-7.6	-7.8
11282394	-7.6	-8.1
107971	-7.5	-8.2
162350 (Isovitexin)	-7.5	-7.9
187808	-7.5	-7.7
441667	-7.5	-7.4
5281377	-7.5	-8.3
11968629	-7.5	-7.6
71621984	-7.5	-8.3
71621987	-7.5	-8.3
85469293	-7.5	-7
137706454	-7.5	-7.7
10095180	-7.4	-8.1
155289778	-7.4	-8.2
5318645	-7.4	-7.2
10095770	-7.2	-7.3
5320844	-7	-8
5491408	-7	-6.8
124034	-6.8	-7.4
5319486	-6.7	-8.3
